# Using a HIV registry to develop accurate estimates for the HIV care cascade – the Singapore experience

**DOI:** 10.1002/jia2.25356

**Published:** 2019-07-26

**Authors:** Zheng Jie Marc Ho, Flora Huang, Chen Seong Wong, Lily Chua, Stefan Ma, Mark I‐Cheng Chen, Vernon J Lee

**Affiliations:** ^1^ Communicable Diseases Division, Ministry of Health Singapore Singapore Singapore; ^2^ National Public Health Unit Singapore Ministry of Health Singapore Singapore Singapore; ^3^ National Centre for Infectious Diseases Singapore Singapore; ^4^ Epidemiology and Disease Control Division Ministry of Health Singapore Singapore Singapore; ^5^ Saw Swee Hock School of Public Health National University of Singapore Singapore Singapore

**Keywords:** HIV, epidemiology, goals, registries, modelling, sampling studies

## Abstract

**Introduction:**

Achieving UNAIDS 90‐90‐90 targets is a crucial step towards ending the AIDS epidemic. Many countries have published estimates of care cascades, although often with methodological limitations. We describe an approach that used the national HIV registry as a starting‐point to determine the HIV care cascade and resulting UNAIDS 90‐90‐90 estimates for Singapore in 2014.

**Methods:**

HIV is a legally notifiable disease in Singapore. The anonymized HIV registry data provided for a back‐calculation model from the European Centre for Disease Prevention and Control to obtain 2014 estimates for the total number of persons living with HIV (PLHIV), and the count in the registry for proportions diagnosed with HIV and linked to care. Using additional data collected for a simple random sample from the registry, outcomes in 2015 and 2016 were ascertained retrospectively to derive proportions for those retained in care, on antiretroviral therapy, and achieved viral suppression. Findings were extrapolated to derive national estimates and UN90‐90‐90 estimates. Bootstrapped samples from the model and sample were used to derive 95% confidence intervals.

**Results:**

An estimated 6900 (95% CI 6650, 7050) persons were living with HIV and AIDS in 2014. Of these, 4948 were diagnosed with HIV, and 4820 had been linked to care. The random sample of 500 persons was further analysed, and of these, 87.2% were retained in care, 84.6% on antiretroviral therapy, and 79.6% had suppressed viral loads. The proportions of HIV‐infected individuals on antiretroviral therapy and achieving viral suppression were 60.7% (95% CI 58.4, 63.6) and 57.1% (95% CI 55.0, 60.5) respectively. The corresponding UNAIDS 90‐90‐90 estimates were 71.7% (95% CI 70.0, 74.2) of all persons diagnosed; 84.6% (95% CI 81.6, 87.4) of diagnosed persons being on antiretroviral therapy, and 94.1% (95% CI 91.6, 96.2) of persons on therapy having achieved viral suppression.

**Conclusions:**

A national HIV registry, alongside back‐calculation and additional data from a sample, can be used to estimate attainment of UNAIDS 90‐90‐90 targets and identify system gaps. The registry had advantages of providing a well‐established, comprehensive capture of diagnosed persons and easily accessible data. The same approach can be used elsewhere if similar data are available.

## 1 Introduction

Combination antiretroviral therapy (ART) has greatly reduced HIV‐associated mortality, morbidity and transmissibility. However, new infections, diagnoses of AIDS and deaths attributable to HIV/AIDS remain high 1.

In 2014, the Joint United Nations Programme on HIV/AIDS (UNAIDS) and the World Health Organization (WHO) proposed the 90‐90‐90 HIV/AIDS treatment goals. These targets were for 90% of all people living with HIV/AIDS (PLHIV) to be diagnosed; 90% of those diagnosed to be started on antiretroviral therapy (ART); and 90% of those on treatment to achieve undetectable HIV viral loads. This ensures that at least 73% of PLHIV would be virally suppressed – a crucial step towards ending the AIDS epidemic 2. Achieving viral suppression involves intermediate steps, otherwise known as the HIV care cascade. Many countries have published estimates of their own cascades, although often with methodological limitations 3.

Singapore is a city‐state in Southeast Asia with a population of 5.6 million in 2016. The first case of HIV was detected in 1985, followed by a rapid rise in the number of newly diagnosed cases, peaking in 2008. Majority of cases are male (10:1 male to female), with highest rates among the 20‐29 and 50‐59 age groups in males and females respectively 4. Although incidence has remained steady since 2012, there are concerns that many remain undiagnosed and present late with AIDS 5. In this paper, we describe a methodology that used the National HIV Registry as a starting‐point to determine the care cascade in Singapore, and the resulting estimates for PLHIV who were diagnosed by end‐2014, using data of a representative sample in 2015 and 2016.

## 2 Methods

### 2.1 The National HIV Registry and overview of study design

Under the Infectious Diseases Act, all diagnosed cases of HIV or AIDS must be notified, by doctors and the central HIV confirmatory laboratory, to the National HIV Registry 6. Started in 1985, this registry is a name‐based system which holds identifiable data for known HIV cases in Singapore, as well as contacts of these cases that were reported to the registry. Only selected registry staff gazetted as public health officers under the Infectious Diseases Act, are allowed to collect epidemiological and clinical information from the national electronic health records system, physical medical records at clinics and hospitals, and by interviewing healthcare providers when necessary 7. More information of the surveillance system can be found in the Supplementary.

In Singapore's context, the spectrum of engagement in HIV care comprises six stages (Table 1), aligned with earlier publications by USA and other countries 8, 9. In addition to stages corresponding to the UNAIDS 90‐90‐90 targets, these included intermediate stages for linkage and retention, which were beneficial for evaluating national healthcare targets. Anonymized national HIV registry data were used for a back‐calculation mathematical model for the first stage, and cross‐sectional data for the second stage. The third stage was ascertained through a retrospective cohort analysis of the entire registry. Additional outcomes for a sample cohort from the registry were obtained retrospectively, by authorized registry staff, to derive data for the last three stages.

**Table 1:** The spectrum of engagement in HIV care

	HIV testing and treatment cascades	UNAIDS 90‐90‐90 targets	Data source	Method
Stage 1	HIV‐infected		Full HIV registry	Back‐calculation mathematical model
Stage 2	HIV‐diagnosed	First 90 = Stage 2/Stage 1		Cross‐sectional analysis
Stage 3	Linked to HIV care			Retrospective cohort analyses
Stage 4	Retained in HIV care		Sample of HIV registry	
Stage 5	On ART	Second 90 = Stage 5/Stage 2		
Stage 6	Suppressed viral load	Third 90 = Stage 6/Stage 5		

ART, antiretroviral therapy; HIV, human immunodeficiency virus; UNAIDS, United Nations Programme on HIV/AIDS.

This analysis was led by the Ministry of Health Singapore, through the Infectious Diseases Act, to understand the current local epidemiology of HIV/AIDS and to update UNAIDS on Singapore's progress 7. Steps taken to ensure confidentiality were in accordance to the Act – after authorized registry staff had collected the necessary cohort data, the dataset was password‐encrypted and identifiers removed prior to further analysis. The entire analysis was conducted within a secured environment under the Ministry and Registry.

### 2.2 Stage 1: persons living with HIV or AIDS

To account for undiagnosed individuals, a model proposed and validated by the European Centre for Disease Prevention and Control (ECDC), Version 1.2.2, was applied to the total number of persons in the HIV registry as of 31 Dec 2016. This was a multi‐stage back‐calculation model that used surveillance data on new HIV and AIDS diagnoses. In brief, it describes HIV progression as a unidirectional flow through different stages of the infection as shown by CD4 counts or occurrence of AIDS events. More details can be found in the manual 10. It was adopted rather than the UNAIDS EPP (Estimation and Projection Package) because the former used routinely collected data on HIV (including CD4 counts and AIDS diagnoses), easily obtainable through the registry. Moreover, EPP required sizes and distributions of sub‐populations (for example intravenous drug users) and assumed that current distributions applied to projected years 11.

Data from cases reported in 2015 and 2016 were included in the modelling to provide more accurate estimates for 2014. Two years’ worth of most recent data were used because confidence intervals for the number of new HIV infections (which in turn would affect the back‐calculation model) was anticipated to be larger when deriving estimates for recent years. Hence, we used cases as of 2014 as a cut‐off. It balanced between having information that was timely enough to be relevant while being sufficiently accurate.

### 2.3 Stage 2 and First 90: diagnosed with HIV

A PLHIV diagnosed with HIV was defined as a person who was diagnosed by 31 December 2014. The First 90 target was thus calculated as the proportion of all diagnosed persons, as captured by the HIV registry as of 31 December 2014, divided by the modelled number of PLHIV (Stage 1).

### 2.4 Stage 3: linked to care

A PLHIV linked to care was defined as a person who was diagnosed and ever had one provider visit for HIV care following a diagnosis by 31 December 2014. This was based on any evidence showing receipt of HIV care collected by public health officers upon notification to the registry, comprising any of the following: (i) CD4 cell count measurement; (ii) HIV viral load measurement; (iii) at least one consult with an infectious diseases specialist; (iv) at least one review by a Medical Social Worker for HIV follow‐up care; or (v) at least one prescription for ART. The proportion was calculated as all persons linked to care according to the HIV registry, divided by the modelled number of PLHIV (Stage 1).

### 2.5 Random sampling of PLHIV (for stages 4 to 6)

To estimate the number of PLHIV retained in care (Stage 4), on antiretroviral therapy (Stage 5), and who had suppressed viral loads (Stage 6), authorized public health officers from the registry purposively collected additional data using a simple random sample of 500 PLHIV from within the HIV registry that were alive based on the National Registry of Births and Deaths as of 31 December 2014 (using national identification numbers). This process was similar to how data are collected upon diagnosis, albeit covering the follow‐up period, and included any evidence of receipt of HIV care, ART prescriptions and viral load results. Persons included in the cohort were not approached. Sampling was necessary because the registry did not routinely collect all clinical treatment data necessary to accurately estimate these latter stages. The additional data were securely stored in the registry and anonymized prior to analysis.

The sample size was based on a confidence level of 95%, a margin of error of 4% and an attrition (left Singapore/followed up overseas or subsequently discovered to have died) of 1.2%. The attrition was assumed to be similar to observations from a previous analysis on an earlier cohort using the same approach [unpublished data].

### 2.6 Stage 4: retained in care

A PLHIV retained in care was defined as a person who was diagnosed and had evidence of having had received HIV care in 2014. The evidence required was the same as that used for Linkage to Care (Stage 3).

The proportion retained in care in the sampled cohort was used to extrapolate the number of PLHIV retained in care in Singapore. The proportion retained in care was then calculated as the extrapolated population divided by the modelled number of PLHIV (Stage 1).

### 2.7 Stage 5 and Second 90: on antiretroviral therapy

A PLHIV on ART was defined as a person who was diagnosed and had a record of an ART prescription in 2014. If they had no records pertaining to ART prescription in 2014, they were considered to be on ART if found to have viral suppression in 2015 or 2016.

Again, the number of PLHIV on ART in Singapore was extrapolated based on the proportion in the sampled cohort, and the corresponding proportion then calculated as the extrapolated population divided by the modelled number of PLHIV (Stage 1). An estimation of the Second 90 target was calculated as the extrapolated population of persons on ART divided by the number of HIV diagnosed persons captured by the HIV registry (Stage 2).

### 2.8 Stage 6 and Third 90: suppressed viral load

A PLHIV with viral suppression was defined as a person who was diagnosed and with a viral load measurement of <200 copies/mL taken after starting ART in 2014. This was based on medical records from 2015 and 2016. This cut‐off was the threshold for virological failure in the US Department of Health and Human Services guidelines, which were widely adhered to locally 12. If multiple records were available, the latest measurement was taken.

The use of two years of data for Stages 5 and 6 was in part an effect of parameters used to derive accurate Stage 1 estimates. Records up to end‐2016 were used as it was the most recent year‐long dataset at the time of analysis. This is regardless of when the cases were actually diagnosed. Furthermore, some patients who were on long‐term treatment and virally suppressed for years may not be tested within the calendar year, hence two years was used to prevent exclusion of these records. If no recent records were available, these persons were assumed to be unsuppressed (that is, worst‐case scenario).

Similar to previous stages, we used the proportion from the sampled cohort to extrapolate the number of PLHIV with viral suppression in Singapore. The proportion that were virally suppressed was then calculated as the extrapolated population divided by the modelled number of PLHIV (Stage 1), with the Third 90 target calculated as the extrapolated population with suppressed viral load divided by the extrapolated population of diagnosed with HIV on ART (Stage 5).

### 2.9 Confidence intervals

To quantify the uncertainty associated with estimates, we used bootstrapped samples from the ECDC HIV modelling tool. We also generated bootstrapped samples from the random sample of 500 patients to estimate the sampling variability of the indicators. From these data, we derived 95% confidence intervals for each stage and the 90‐90‐90 targets.

## 3 Results

### 3.1 Stage 1 to 3

**Figure 1:**
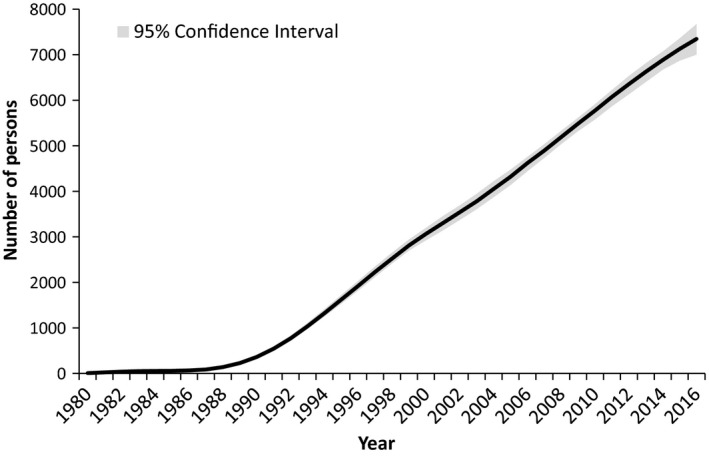
**Number of persons living with HIV in Singapore.** As estimated through the European Centre for Disease Prevention and Control model.

**Figure 2:**
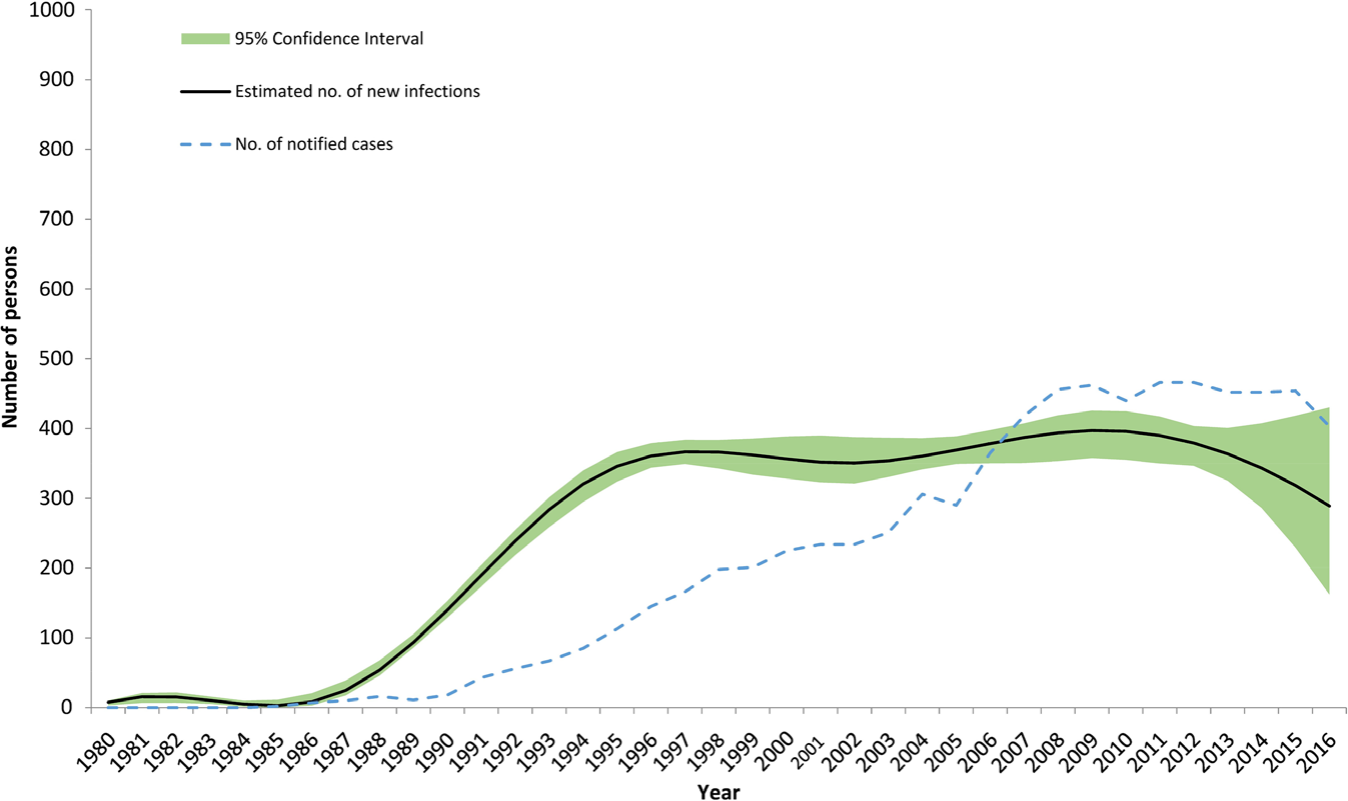
**Number of new HIV infections in Singapore from 1980 to 2016.** The dashed line indicates the number of cases notified to the HIV registry per year. The black line is the number of new infections as estimated through the European Centre for Disease Prevention and Control model, with the shaded portion as the corresponding 95%CI.

**Figure 3:**
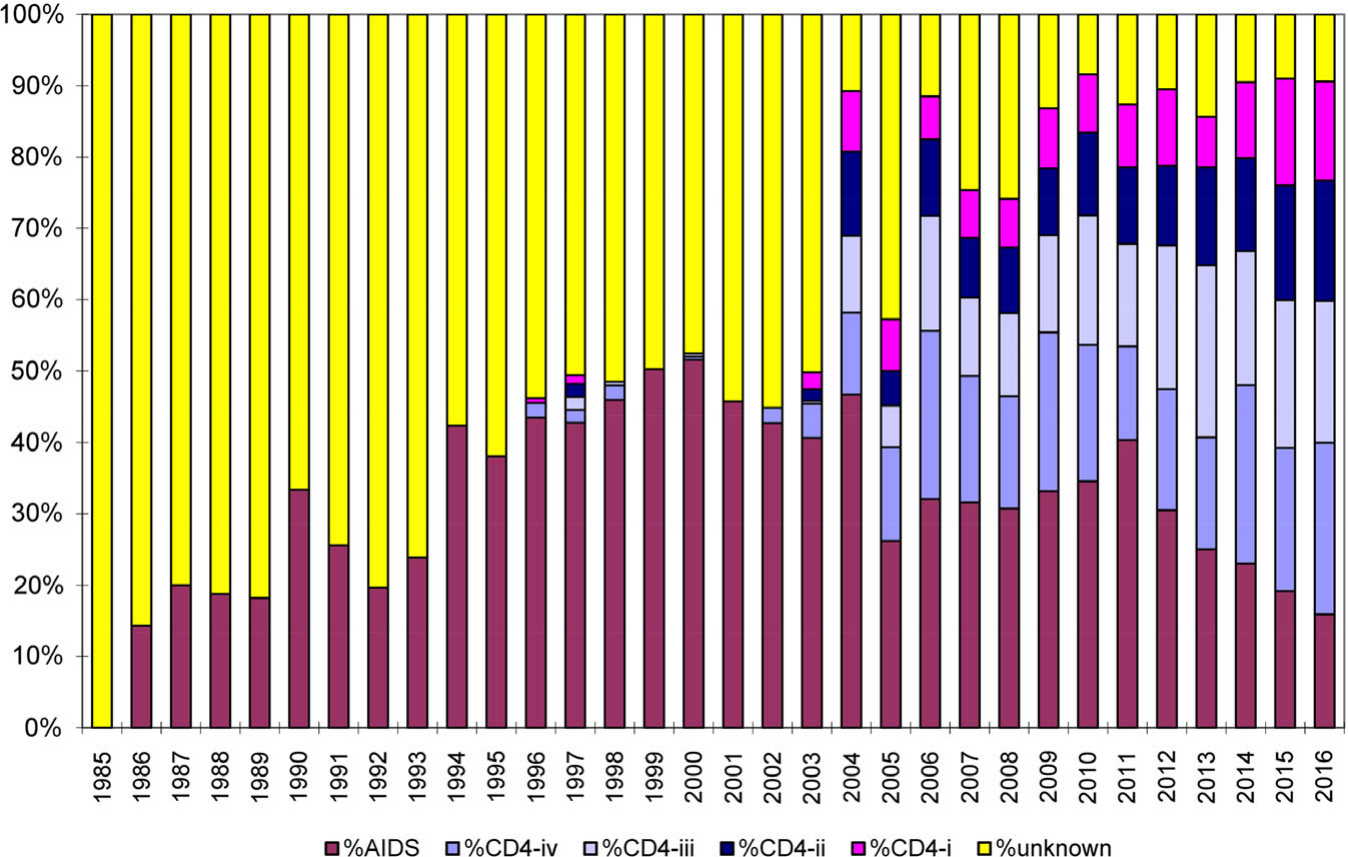
**Proportion of new AIDS and HIV cases per year in Singapore, stratified by CD4 counts.** These were used as inputs to the European Centre for Disease Prevention and Control model. %AIDS represents the proportion of new cases with concurrent HIV and AIDS diagnoses. %HIV CD‐i to CD‐iv represents the proportion of new HIV diagnoses with CD4 counts of (i) ≥500 cells/mm^3^, (ii) 350‐499 cells/mm^3^, (iii) 200‐349 cells/mm^3^, and (iv) <200 cells/mm^3^ respectively and without a concurrent diagnosis of AIDS.

Based on the HIV registry, of the 6685 persons who had been ever diagnosed with HIV as of 31 December 2014, 4948 were alive based on the Registry of Births and Deaths. Including undiagnosed cases, the model estimated 6900 (95% CI 6650, 7050) PLHIV (Figure 1), including 340 new HIV infections in 2014 (Figure 2). The estimated number of PLHIV in Singapore has been increasing linearly since the 1990s, with plateauing in the estimated number of new cases from the mid‐1990s. Although estimates also showed a likely decline in the number of new HIV infections from 2010, confidence intervals were wide and more years of data would be needed for certainty. In comparison, the confidence intervals for the total number of PLHIV in 2016 were only slightly wider (95% CI 7000, 7650). In 2009, 55.4% of new cases presented with either CD4 counts of <200 cells/mm^3^ or clinical AIDS, compared to 40.0% in 2016 (Figure 3).

A total of 4820 persons were linked to HIV care by the end of 2014, representing 97.4% of those diagnosed, and 69.9% (95% CI 68.2, 72.3) of the total PLHIV population.

### 3.2 Random sampling and findings for Stages 4 to 6

**Figure 4:**
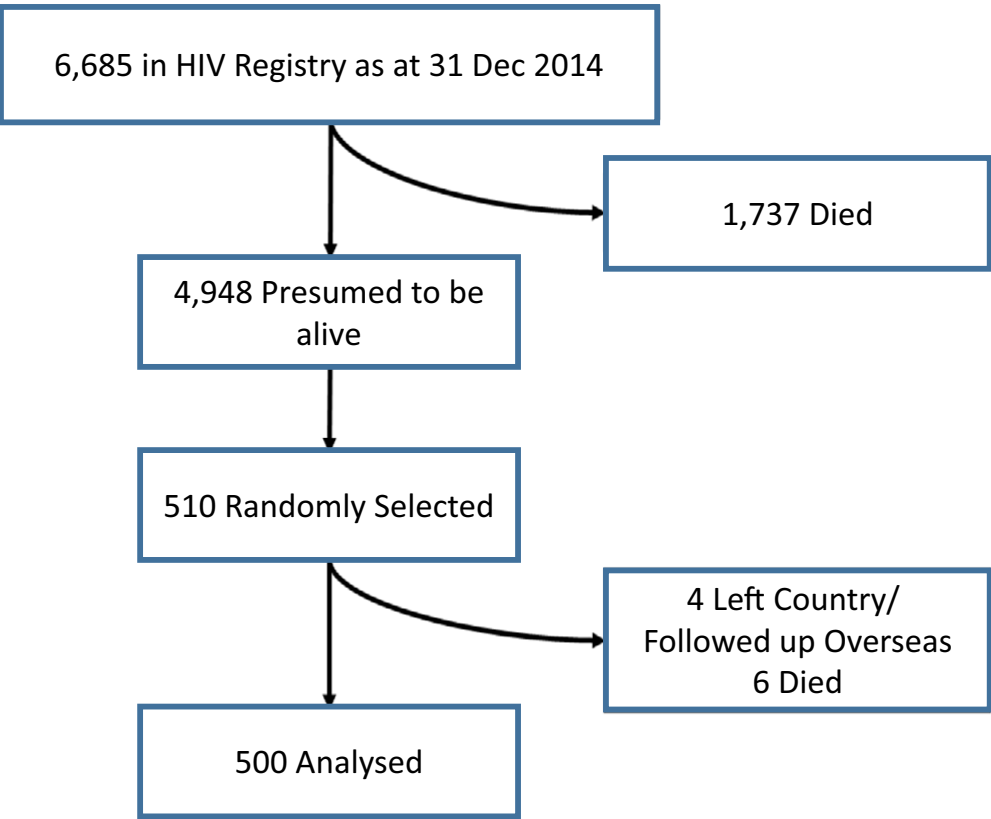
**Sampled population to estimate stages 4 to 6.**

A total of 510 persons were randomly selected. Of these, four were excluded as they were found to have left the country or transferred their care to an overseas provider. Another six were found to have passed away. The final sample size of 500 cases was analysed (Figure 4). Demographics of the sample were compared with that of the entire HIV registry and found to be comparable for age, gender, race and mode of transmission (*p* > 0.05; Table S1).

Of the 500 sampled cases, 436 (87.2%) were retained in HIV care, 423 (84.6%) were on ART, and 398 (79.6%) had suppressed viral loads. Given 4948 persons were presumed to be alive in the HIV registry, we hence extrapolated that 4315, 4186 and 3939 persons were in the corresponding stages.

### 3.3 UNAIDS 90‐90‐90 targets

**Figure 5:**
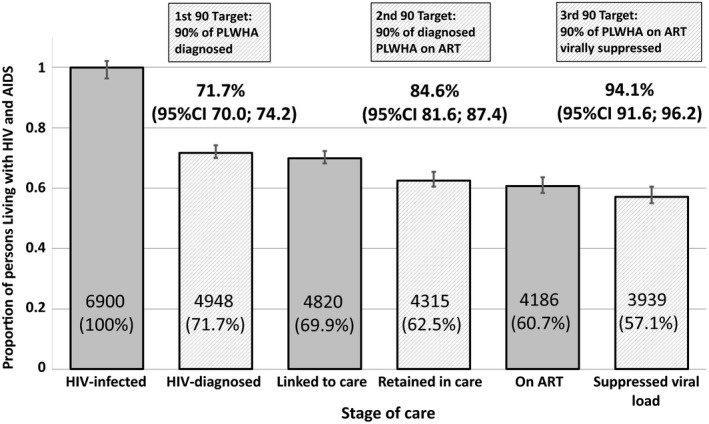
**Singapore's progress towards UNAIDS 90‐90‐90 in 2014.** Numbers within bars are the estimated total number of persons in Singapore in each category, and the accompanying percentages are the proportion of all HIV‐infected persons in Singapore in each category.

These findings translated to an estimated 71.7% (95% CI 70.0, 74.2) of all PLHIV in Singapore diagnosed (First 90); 84.6% (95% CI 81.6, 87.4) of those diagnosed being placed on ART (Second 90); and 94.1% (95% CI 91.6, 96.2) of those on ART achieving viral suppression (<200 copies/mL) (Third 90). (Figure 5) Correspondingly, the proportions of all HIV‐infected individuals on ART and achieving viral suppression were 60.7% (95% CI 58.4, 63.6) and 57.1% (95% CI 55.0, 60.5) respectively. Sensitivity analyses (Supplementary Material, Table S3, and Figure S1) suggest that these estimates would be somewhat sensitive to assumptions about under‐reporting as well as the quality of data and definitions used for the proportion of diagnosed PLHIV on ART; with other sources of uncertainty being less influential.

## 4 Discussion

We describe an approach using the National HIV Registry to derive estimates for the HIV care cascade, in the context of UNAIDS 90‐90‐90 targets. While Singapore had reached the third target (94%), ensuring diagnosed PLHIV were on ART (85%) and reducing under‐diagnosis (72%) could be improved.

### 4.1 Singapore's 90‐90‐90 estimates

The success of well‐performing countries has been attributed to factors such as epidemic stage, population demographics, healthcare infrastructure and treatment costs 3, 9. Singapore's 90‐90‐90 results were generally favourable, possibly due to an accessible healthcare system, availability of financial subsidies for ART and adherence to international treatment guidelines.

However, under‐diagnosis of early HIV illness remains a concern. As shown in Figure 3, a substantial proportion still present with late stage infections. Underlying reasons include reluctance to test due to stigma and discrimination, lack of awareness of testing benefits, low risk perception of HIV exposure and fear of undesirable socio‐economic consequences 13, 14, 15. Late presentation was previously also associated with sociodemographic factors such as older age, singlehood and working in non‐professional occupations 16. Closing this gap would thus require differentiated strategies that account for heterogeneity among those most at risk, and specifically reach key populations. For example, harnessing innovations such as home and self‐testing, mobile or e‐health services, as well as social network, partner counselling and referral services have been shown to be effective for men who have sex with men 17. Rapid voluntary counselling and testing has also been shown to significantly increase uptake of testing and return for results 18. These are currently available at anonymous test sites in primary care and community‐based organizations in Singapore, and should be further expanded 19. Self‐testing is currently not available locally, although studies on their utility and local acceptability are promising, especially if user friendliness and linkage to care can be adequately addressed 20.

Intermediate steps suggest that retention in care could also be improved, alongside better understanding of complex factors underpinning ART uptake and adherence. These include personal motivations, and system barriers and enablers that impact cost and accessibility 21, 22. Singapore has a multi‐payer healthcare financing framework involving a mix of patient co‐payment, government subsidies, healthcare savings, health insurance and a safety net 23. Previous local studies found that the high cost of ART was a concern, and that stigma and shame persisted while receiving medical care 24, 25, 26. Singapore has been working towards increasing access to subsidies, improving public awareness and education through community organizations, and reducing stigma and discrimination at workplaces.

Population level disparities in retention, access to ART and virological suppression have also been found in other settings, including countries in South‐East and East Asia, where facilities face challenges in providing care for a growing number of PLHIV in resource‐limited settings 27, 28. However, sex, race as well as other demographic and socioeconomic disparities in HIV care exist even in developed economies 29, 30, 31. It demonstrates the complexities of chronic care for vulnerable groups disproportionately affected by HIV, and resulting inequities at every step of the care cascade. Areas such as clinical infrastructure, drug supplies, access to reliable testing and adherence counselling also need to keep up with better survival rates. With an increasing number of elderly PLHIV, integration with chronic disease services would be important for holistic care 32. This would also address concerns that the single disease‐centric approach of the UNAIDS 90‐90‐90 strategy may jeopardise funding for other much‐needed health programmes 33.

### 4.2 Deriving accurate 90‐90‐90 estimates

This study also touches on the global challenge of how to accurately and consistently derive estimates to monitor countries’ progress towards UNAIDS 90‐90‐90. Two reviews described the range of approaches undertaken elsewhere, from cumulative cross sections to longitudinal studies and varied combinations of the two 3, 9. Information sources also varied. For example, some calculated prevalence using population‐based surveillance, while others depended on UNAIDS estimates, data from subpopulations or national consensus. Many did not clearly mention their classification definitions, and unstandardized criteria for viral load data meant reliance on indirect sources resulting in underestimates (for example only testing non‐adherent cases or those showing drug resistance) or overestimates (for example only testing cases still on clinical follow‐up). Cut‐offs for viral suppression also ranged from <40 RNA copies/mL in Rwanda and Tanzania to <1000 RNA copies/mL in Russia 9. Such wide heterogeneity has made direct comparisons difficult.

The most accurate approach would be for all necessary data to be drawn from electronic health records. This is unfortunately not currently available for many countries, including Singapore, especially when it involves data from the private sector. We chose the National HIV registry as the start point given that it was well‐established, comprehensive and provided easily accessible data. Once we had developed locally appropriate definitions, it was straightforward to extract the necessary information from registry and clinical records.

The ECDC model adopted had limitations – by depending on data from case reports, underreporting and incomplete documentation could lead to underestimates for HIV incidence and total number of PLHIV 10. Improved approaches, such as emerging technologies, may help. For instance, if actual transmission dates could be derived (for example using molecular clock calculations), it may allow for more sophisticated back‐calculation methods.

Next, although notification of new cases was mandatory, the HIV registry lacked clinical data across time. Many large public hospitals in Singapore maintained individual databases, and some had been contributing to regional cohort studies. However, these were not nationally representative, and the extent of data captured varied 34. These led to reports suggesting that Singapore had achieved all 90‐90‐90 targets, whereas our findings suggest otherwise 35. At the same time, our approach of collecting detailed data from a sample of the registry is still not ideal. Its accuracy depends on sample size and the comprehensiveness of clinical records kept. A two‐year follow‐up period was an effect of parameters used to derive accurate Stage 1 estimates and to account for individuals who may not be tested within the same calendar year. However, given that only the most recent viral load was considered, this was unlikely to have resulted in overestimates. Similar estimates when obtained when only results for 2015 were used (Table S2).

Data obtained from a disease registry or sampling of cases may also contain errors. When we collected sample data, ten cases had incomplete data for outcomes – four had left the country and six had incorrect survival status recorded in the HIV registry. Countries that adopt a similar approach may encounter similar data accuracy issues, and such attrition would need to be accounted for when determining adequate sample sizes. For our analysis, we censored these ten persons in the final sample count. We chose not to extrapolate and adjust estimates for the rest of the cascade given that this number was small (<2%) and its impact would thus be limited. However, in situations where incomplete data are larger, more conservative approaches may be warranted. This includes assuming a worst‐case scenario (for example labelling all as untreated), or extrapolating estimates from cases with complete data to those without. Moreover, PLHIV who had achieved viral suppression in 2015 and 2016 were all assumed to be on ART. We felt that this was appropriate since ART is generally required to induce viral suppression, and laboratory records of viral load testing are likely more complete than clinical documentation of ART prescriptions.

Moving forward, a single consolidated database or standardization and better integration of existing databases may help provide more comprehensive capture and reduce resources required for sampling. Although Singapore has a national electronic health record system, it currently mainly covers data from public healthcare institutions. Private healthcare provides substantial clinical care and laboratory services, and it may be possible to directly obtain more accurate information on Singapore's care cascade once these are also available on the national system.

Regardless, we believe the approach described balances practicability and accuracy, and is thus an option that other countries with reasonably complete national HIV registries may wish to adopt. This approach can and will be used again in future years to monitor Singapore's progress towards UNAIDS 90‐90‐90 targets. Consistency would also allow future look back exercises to evaluate accuracy of the overall methodology. Furthermore, by using adequately‐sized random samples of cases stratified by geographical regions or risk groups, it opens opportunities for within‐country estimates that may help guide public health policies in HIV prevention and control.

## 5 Conclusions

A national HIV registry, alongside back‐calculation and additional direct data collection for a sample, can be used to estimate attainment of UNAIDS 90‐90‐90 targets and identify system gaps in the HIV care cascade. The method provided an overarching view of national progress in HIV control for Singapore, and can potentially be used in other jurisdictions where similar data are available.

## Competing interests

The authors have no conflicts of interest to declare.

## Authors’ contributions

Z.J.M.H, F.H., M.I.C.C. and V.J.L. designed the study. F.H. supervised the data collection. F.H., L.C., S.M. and M.I.C.C. performed the analysis. Z.J.M.H. led the writing of the manuscript. C.S.W. provided domain knowledge and also co‐wrote several key sections. S.M., M.I.C.C. and V.J.L. provided oversight for the direction of the manuscript and made critical edits. All authors have read and approved the final manuscript.

## Supporting information

**Table S1.** Demographic characteristics of the sample as compared to all diagnosed PLHIV in the National HIV Registry as of end‐2014.
**Table S2.** Estimates for PLHIV who were on ART and who achieved viral suppression when only records from 2015 were considered.
**Table S3.** Parameters ranges used in one‐factor‐at‐a‐time sensitivity analyses.
**Figure S1.** Sensitivity analyses of factors influencing estimates for proportion of PLHIV are (i) diagnosed [*D*], (ii) on ART [*T*] and (iii) virally suppressed [*S*].
