# Supplementary material for: Using a HIV registry to develop accurate estimates for the HIV care cascade – the Singapore experience
Source: J Int AIDS Soc. 2019 Jul 26;22(7):e25356. doi: 10.1002/jia2.25356 (PMC6658839; doi:10.1002/jia2.25356)
Supplement: Supplementary file 2 — Table S1. Demographic characteristics of the sample as compared to all diagnosed PLHIV in the National HIV Registry as of end‐2014. Table S2. Estimates for PLHIV who were on ART and who achieved viral suppression when only records from 2015 were considered. Table S3. Parameters ranges used in one‐factor‐at‐a‐time sensitivity analyses. Figure S1. Sensitivity analyses of factors influencing estimates for proportion of PLHIV are (i) diagnosed [D], (ii) on ART [T] and (iii) virally suppressed [S]. [file JIA2-22-e25356-s002.docx]

**SUPPORTING INFORMATION**

**HIV Surveillance in Singapore**

Singapore replies on dual notifications to the National HIV registry, which was set up and maintained by the Ministry of Health after the first HIV case was detected in 1985. The Infectious Diseases Act, which came into force in 1977, mandates that all laboratories and doctors must notify the Ministry of persons infected with one or more of a selected list of infectious diseases of public health importance to Singapore, to ensure complete and timely reporting and ensure that public health actions can be taken if necessary. HIV needs to be notified within 72 hours of diagnosis.

Presently, an IT system called the National Public Health Unit Registry System (NPHURS) is used to record the data. NPHURS acts as a central and confidential repository of name-based HIV and AIDS cases that were notified under the Infectious Diseases Act, for the purposes of public health surveillance. The current system was fully functional in 2012, used to replace an older system, and is hosted at the National Public Health & Epidemiology Unit (NPHEU) of the National Centre for Infectious Diseases Singapore, which acts on behalf of the Ministry of Health. The NPHEU is also where associated HIV activities, such as contact tracing, is carried out.

Public health officers, under the Infectious Diseases Act, collect further details information using a standard epidemiological surveillance form. They record details at the time of HIV diagnosis using data from the national electronic health records system, physical medical records at clinics and hospitals, and by interviewing healthcare providers when necessary. The form comprises biodata, risk factors, clinical status, relevant laboratory investigations (Western Blot and / or Viral Load), linkage / enrolment into HIV care, death status, as well as immune status. Linkage to care was defined as any evidence of the following: (i) CD4 cell count measurement; (ii) HIV viral load measurement; (iii) at least one consult with an infectious diseases specialist; (iv) at least one review by a Medical Social Worker for HIV follow-up care; or (v) at least one prescription for ART. The NPHURS also has a link to the National Registry of Birth and Deaths maintained by the Immigrations and Checkpoints Authority of Singapore.

**Supplementary Table 1**. **Demographic characteristics of the sample as compared to all diagnosed PLHIV in the National HIV Registry as of end-2014**

|  | **All diagnosed PLHIV** | | **Sample** | | **Effect size** |
| --- | --- | --- | --- | --- | --- |
|  | **No.** | **%** | **No.** | **%** |  |
| **Age Group** | | | | | |
| **<25 y.o.** | 144 | 2.9% | 14 | 2.8% | 0.0066 |
| **25 – 34 y.o.** | 708 | 14.3% | 64 | 12.8% | 0.0441 |
| **35 – 44 y.o.** | 1266 | 25.6% | 139 | 27.8% | -0.0501 |
| **45 – 54 y.o.** | 1540 | 31.1% | 149 | 29.8% | 0.0288 |
| **55+ y.o.** | 1290 | 26.1% | 134 | 26.8% | -0.0165 |
| **Total** | 4948 | 100% | 500 | 100% |  |
| **Gender** | | | | | |
| **Male** | 4482 | 90.6% | 448 | 89.6% | 0.0329 |
| **Female** | 466 | 9.4% | 52 | 10.4% | -0.0329 |
| **Total** | 4948 | 100% | 500 | 100.0% |  |
| **Race** | | | | | |
| **Chinese** | 3818 | 77.2% | 383 | 76.6% | 0.0133 |
| **Malay** | 708 | 14.3% | 67 | 13.4% | 0.0263 |
| **Indian** | 255 | 5.2% | 28 | 5.6% | -0.0198 |
| **Others** | 167 | 3.4% | 22 | 4.4% | -0.0531 |
| **Total** | 4948 | 100% | 500 | 100% |  |
| **Mode of Transmission** | | | | | |
| **Heterosexual** | 2643 | 53.4% | 273 | 54.6% | -0.0238 |
| **Homosexual & Bisexual** | 2102 | 42.5% | 212 | 42.4% | 0.0017 |
| **Others** | 203 | 4.1% | 15 | 3.0% | 0.0598 |
| **Total** | 4948 | 100% | 500 | 100% |  |

**Chi-square goodness-of-fit test: p>0.05 for all variables**

**Supplementary Table 2**. **Estimates for PLHIV who were on ART and who achieved viral suppression when only records from 2015 were considered.**

| **On ART** | **2015 Records Only** | **2015 and 2016 Records** | **Difference** |
| --- | --- | --- | --- |
| **Sample proportion** | 0.824 | 0.846 | 0.022 |
| **95% CI (asymptotic)** | 0.7906, 0.8574 | 0.8144, 0.8776 | -0.024, 0.068 |
| **z-value** | 0.9 | | |
| **p-value** | 0.3487 (2-tailed test, Significance level = 0.05) | | |
| **Viral Suppression** | **2015 Records Only** | **2015 and 2016 Records** | **Difference** |
| **Sample proportion** | 0.756 | 0.796 | 0.04 |
| **95% CI (asymptotic)** | 0.7184, 0.7936 | 0.7607, 0.8313 | -0.0117, 0.0917 |
| **z-value** | 1.5 | | |
| **p-value** | 0.1293 (2-tailed test, Significance level = 0.05) | | |

**Sensitivity analyses**

Various stages in the HIV care cascade depend on other estimates, particularly when later stages are expressed as a proportion of all PLHIV, rather than as a proportion of cases in the immediately preceding stage. Such estimates potentially suffer from amplification of statistical uncertainty and any biases in values derived for earlier stages in the cascade.

For instance, the proportion of PLHIV who are on ART, *T*, is a product of the proportion of PLHIV who are diagnosed [*D*], and the proportion of diagnosed PLHIV who are on ART [*A*], i.e. *T = D × A*. Likewise, the proportion of PLHIV who are virally suppressed, *S*, is a product of *D, A,* and also *V*, the proportion of PLHIV on ART who are virally suppressed, i.e. *S = D × A × V*. The terms *D, A* and *V* are in turn subject to uncertainty from the factors these estimates are based on. *D* is the difference between the number of cases reported to the HIV registry [*r*] and the number who have died [*m*], divided by the number estimated to be alive with HIV from the ECDC model [h], i.e. *D = (r – m) / h.* In turn, *A* is the number traced who were on ART [*p*] divided by the total number traced and analysed [*n*], i.e. *A = p / n;* and *V* is the number traced as virally suppressed at <200 copies / ml [*c*] divided by *p*, i.e. *V = c / p* (while assuming that, in addition to those prescribed ART who did not achieve virological suppression, all who achieved virological suppression had also received ART regardless whether the prescription was captured).

Approach

Given the various sources of uncertainty, we hence conducted a simple sensitivity analyses. We assessed how three key measures deviated from their base case estimates under different assumptions, namely:

- The proportion of PLHIV who are diagnosed, *D*
- The proportion of PLHIV who are on ART, *T*
- The proportion of PLHIV who are virally suppressed, *S*

We firstly identified all factors used to derive the three estimates, and for each factor, we then performed sensitivity analyses, one factor at a time. We framed uncertainty in three different ways.

Firstly, to assess amplification of uncertainty in the proportions of interest, we varied the factors used to derive each proportion by up to a standard +/-20% of their base case value.

Secondly, the proportions derived are subject statistical uncertainty denoted by the corresponding confidence intervals. We hence computed the variation which occurs when using the lower and upper bounds of the 95% confidence intervals for each of the component factors.

Finally, we were especially concerned with biases that could potentially cause the true proportion to be substantially lower than the base case estimate, and thus defined a set of “pessimistic scenarios” for each factor as specified in Supplementary Table 3. While all laboratories and doctors are legally mandated to notify HIV diagnoses to the Ministry of Health, exemption is granted to designated anonymous test sites. These sites do not record the identity of cases testing HIV positive but collect basic data, including the intent to seek treatment outside of Singapore. Such intent was self-reported by 17 newly diagnosed cases from 2014 to 2015, and the ratio of this to the 911 cases diagnosed from 2014 to 2015 was taken as potential indicator of an additional fraction of people living with HIV but not reported to the HIV registry. Also, we note that our process for matching the HIV registry to the Registry of Births and Deaths had under-ascertained deaths. Of 510 individuals originally assumed to be alive, 6 were determined after further investigation to have died. We hence applied a correction factor to inflate *m* by 1.2%. Next, given we defined being on ART as having one or more prescriptions within a 1-year window, we considered the impact of potential treatment interruption in the same time frame. A clinical cohort study which included a substantial number of observations from Singapore found that only 87.9% of those initiating treatment had continued on their first-line regiment after 1 year of follow-up (although notably some would re-start ART with second-line treatment regimens) [Ref S1]. This was used to adjust downwards the proportion of diagnosed PLHIV on ART. Finally, a separate study on the same cohort found a virological failure rate of 3.6 events per 100 patients per year in the group of treated patients from high income countries, and this was used to adjust downwards the proportion of PLHIV with virological suppression [Ref S2].

Findings

Our sensitivity analyses showed that all three measures (the proportion of PLHIV who are diagnosed [*D*], on ART [*T*], and virally suppressed [*S*], Supplementary Figures S1A, B and C respectively) did not vary much by the range of values assumed for the number who had died [*m*]. However, they were similarly rather sensitive to variability in estimates for the number living with HIV [*h*], % of diagnosed PLHIV on ART [*A*], and % on ART are suppressed [*V*]. The variation based on the upper and lower bounds of the 95% confidence intervals of all component factors did not exceed 5% for any of the measures. Notably, the 95% confidence intervals for factors derived from the sample of 500 cases [*A* and *V*] caused variations of similar magnitude to those observed for the number living with HIV as estimated from the ECDC model [*h*]. The “pessimistic scenarios” mostly did not cause a substantial deviation from base case estimates. The exception was the pessimistic scenario assumed for the proportion of diagnosed PLHIV on ART [*A*], where we estimated that only 53.3% of PLHIV were on ART, and only 47.2% were virally suppressed (compared to 60.7% and 57.1% respectively for our base case estimates).

Additional Notes
Several limitations to our sensitivity analyses should be highlighted. Firstly, we could not incorporate levels of uncertainty directly into the ECDC modelling package when estimating the number living with HIV [*h*]. This would have necessitated complex and largely arbitrary choices, for instance, about how under-reporting influences the distribution of notified cases with different CD4 counts and who are at various stages HIV infection. Secondly, the pessimistic scenarios chosen, while having some basis, are admittedly arbitrary. Finally, we did not combine the uncertainty from different factors but only conducted one-factor-at-a-time sensitivity analyses given the distributions of uncertainty in these factors was unknown.

In spite of our limitations, the exercise was useful, and identified a key area for improvement, namely more accurate data on the proportion of diagnosed PLHIV on ART. This could come from detailed clinical cohorts that contain more accurate and detailed data on initiation and interruption of ART.

**Supplementary References**

Reference S1 Martinez-Vega R, De La Mata NL, Kurmarasamy N, et al. Durability of antiretroviral therapy regimens and determinants for change in HIV-1-infected patients in the TREAT Asia HIV Observational Database (TAHOD-LITE). Antivir Ther. 2018;23(2):167-178.

Reference S2 Jung IY, Boettiger D, Wong WW, et al. The treatment outcomes of antiretroviral substitutions in routine clinical settings in Asia; data from the TREAT Asia HIV Observational Database (TAHOD). J Int AIDS Soc. 2017 Dec;20(4).

**Supplementary Table 3**. **Parameters ranges used in one-factor-at-a-time sensitivity analyses.**

| **Parameter** | **Symbol** | **Base case (95%CI)** | **% change over base case in pessimistic scenario where applicable** | |
| --- | --- | --- | --- | --- |
|  |  |  | **Value** | **Rationale** |
| ***No. living with HIV^1^*** | *h* | 6900 (6650, 7050) | +1.9% | Based on ratio of newly diagnosed anonymous test site HIV cases who expressed intent to seek care overseas to no. of HIV cases reported in 2014 to 2015 (17:911 respectively). |
| ***No. reported to HIV registry^2^*** | *r* | 6685 | - |  |
| ***No. in HIV registry who have died^3^*** | *m* | 1737 | +1.2% | Applies additional percentage found to have died in traced sample (6/510) to account for under-reporting of deaths |
| **No. traced and analyzed^4^** | *n* | 500 | - | Considered under the factors examined below, since *A = p / n* and *V = c / p* (where *A* and *V* are the 2^nd^ and 3^rd^ UNAIDS 90-90-90 targets respectively)*.* |
| **No. traced on ART^4^** | *p* | 423 | - |  |
| **No. traced with viral load <200 copies / ml^4^** | *c* | 398 | - |  |
| **% of diagnosed PLHIV on ART^5^** | *A* | 84.6 (81.6, 87.4) | -12.1% | Clinical cohort (including patients from Singapore) where probability of continuing first-line ART regimen at 1 year of follow-up was 87.9% [Ref 1]. |
| **% of PLHIV on ART vir-ologically suppressed^5^** | *V* | 94.1 (91.6, 96.2) | -3.6% | Clinical cohort (including patients from Singapore) where virologic failure rate was 3.6 per 100 patients per year for the high income country group [Ref 2]. |
| ^†^95% confidence intervals generated using a Poisson distribution with a mean count of 1737  ^1^Estimated using the ECDC model with the HIV registry data  ^2^Direct from HIV registry  ^3^Matching of HIV registry with Registry of Births and Deaths  ^4^Sample of HIV cases that we traced and then further analyzed for outcome data, and the numbers traced to be on ART and with viral suppression defined as <200 copies /ml  ^5^Proportions corresponding to 2^nd^ and 3^rd^ UNAIDS 90-90-90 targets, where *A = p / n and V = c / p*; formula for the 1^st^ target, *D,* is *D = (r – m) / h*  Ref 1: Martinez-Vega R, et al. Durability of antiretroviral therapy regimens and determinants for change in HIV-1-infected patients in the TREAT Asia HIV Observational Database (TAHOD-LITE). Antivir Ther. 2018;23(2):167-178.  Ref 2: Jung IY, et al. The treatment outcomes of antiretroviral substitutions in routine clinical settings in Asia; data from the TREAT Asia HIV Observational Database (TAHOD). J Int AIDS Soc. 2017 Dec;20(4). | | | | |

**Supplementary Figure 1** – **Sensitivity analyses of factors influencing estimates for proportion of PLHIV are (i) diagnosed [*D*], (ii) on ART [*T*], and (iii) virally suppressed [*S*].** In all panels, the base case estimate is shown as a dashed vertical line, while horizontal solid lines with whiskers indicate the deviation caused by using the upper and lower 95% confidence intervals for the factor under consideration, with the respective coloured bars giving the effect for increasing or decreasing that factor by 10% and 20%.
